# A Preliminary Study on the Relationship Between High-Resolution Computed Tomography and Pulmonary Function in People at Risk of Developing Chronic Obstructive Pulmonary Disease

**DOI:** 10.3389/fmed.2022.855640

**Published:** 2022-05-04

**Authors:** Rui Lv, Mengyao Xie, Huaqian Jin, Pingping Shu, Mingli Ouyang, Yanmao Wang, Dan Yao, Lehe Yang, Xiaoying Huang, Yiran Wang

**Affiliations:** ^1^Key Laboratory of Respiratory Circulation, Division of Pulmonary Medicine, The First Affiliated Hospital, Wenzhou Medical University, Wenzhou, China; ^2^Department of Intensive Care Unit, Ningbo First Hospital, Ningbo, China

**Keywords:** COPD, HRCT, pulmonary function, air trapping index, mean expiratory lung density

## Abstract

**Objectives:**

Patients with chronic obstructive pulmonary disease (COPD) have high morbidity and mortality, the opportunity to carry out a thoracic high-resolution CT (HRCT) scan may increase the possibility to identify the group at risk of disease. The aim of our study was to explore the differences in HRCT emphysema parameters, air trapping parameters, and lung density parameters between high and low-risk patients of COPD and evaluate their correlation with pulmonary function parameters.

**Methods:**

In this retrospective, single-center cohort study, we enrolled outpatients from the Physical Examination Center and Respiratory Medicine of The First Affiliated Hospital of Wenzhou Medical University. The patients who were ≥ 40 years-old, had chronic cough or sputum production, and/or had exposure to risk factors for the disease and had not reached the diagnostic criteria is considered people at risk of COPD. They were divided into low-risk group and high-risk group according to FEV_1_/FVC ≥ 80% and 80%>FEV_1_/FVC ≥ 70%. Data on clinical characteristics, clinical symptom score, pulmonary function, and HRCT were recorded.

**Results:**

72 COPD high-risk patients and 86 COPD low-risk patients were enrolled in the study, and the air trapping index of left, right, and bilateral lungs of the high-risk group were higher than those of the low-risk group. However, the result of mean expiratory lung density was opposite. The emphysema index of left, right, and bilateral lungs were negatively correlated with FEV_1_/FVC (correlation coefficients were -0.33, -0.22, -0.26). Consistently, the air trapping index of left and right lungs and bilateral lungs were negatively correlated with FEV_1_/FVC (correlation coefficients were -0.33, -0.23, -0.28). Additionally, the mean expiratory lung density of left and right lungs and bilateral lungs were positively correlated with FEV_1_/FVC (correlation coefficients were 0.31, 0.25, 0.29).

**Conclusion:**

The emphysema index, air trapping index and the mean expiratory lung density shows significantly positive correlation with FEV_1_/FVC which can be used to assess the pulmonary function status of people at risk of COPD and provide a useful supplement for the early and comprehensive assessment of the disease.

## Introduction

Chronic obstructive pulmonary disease (COPD) is a common, preventable and treatable disease that is characterized by persistent airflow limitation that is usually progressive and not completely reversible (1). The late stage of the disease is often accompanied by systemic multi-system chronic diseases, including cardiovascular diseases, metabolic syndrome, osteoporosis, depression, anxiety, and lung cancer, which contribute to the overall severity in patients (2, 3).

The clinical diagnosis of COPD is primarily based on pulmonary function tests (PFTs) (4), but some constraints remain in detecting early changes in lung structure or function. As a general function test, the accurate assessment of local pulmonary function damage is difficult. Also, it has been reported in the literature that clinical symptoms will be present or changes in PFTs will appear when more than 30% of the total lung parenchyma has been destroyed (5). Although the forced expiratory volume in one second percentage (FEV_1_%) may be unchanged, there can be significant changes in imaging findings (6). The Global Strategy for the Diagnosis, Management, and Prevention of Chronic Obstructive Pulmonary Disease 2020 report suggests that further in-depth research is needed to study those patients without evidence of airflow limitation but who have evidence of structural lung disease upon chest imaging, such as emphysema, that is consistent with what is found in patients with COPD (7).

Computed tomography (CT) is the modality of choice for the imaging characterization of COPD patients, and it can be used to describe the changes in lung parenchyma in patients with COPD (8). In the past years, with the rapid development of high-resolution computed tomography (HRCT) technology and continuous development of image post-processing and reconstruction techniques, CT imaging has been recognized as an important method for the assessment of COPD (9). The emphysema index (EI) (10, 11), air trapping index (ATI) (12–14), and mean lung density (MLD) (15, 16) are the most common functional imaging parameters. However, these have rarely been evaluated in a population of patients at risk for developing COPD.

The primary aim of our research was therefore to evaluate the differences in HRCT emphysema parameters, air trapping parameters, and lung density parameters between patients with high and low risk of developing COPD, and to investigate the relationships between CT metrics and pulmonary function parameters.

## Materials and Methods

This retrospective and single-center cohort study was carried out at The First Affiliated Hospital of Wenzhou Medical University in China. Ethical approval was obtained, and all participants consented to be included in the trial.

### Recruitment and Enrollment

**FIGURE 1:**
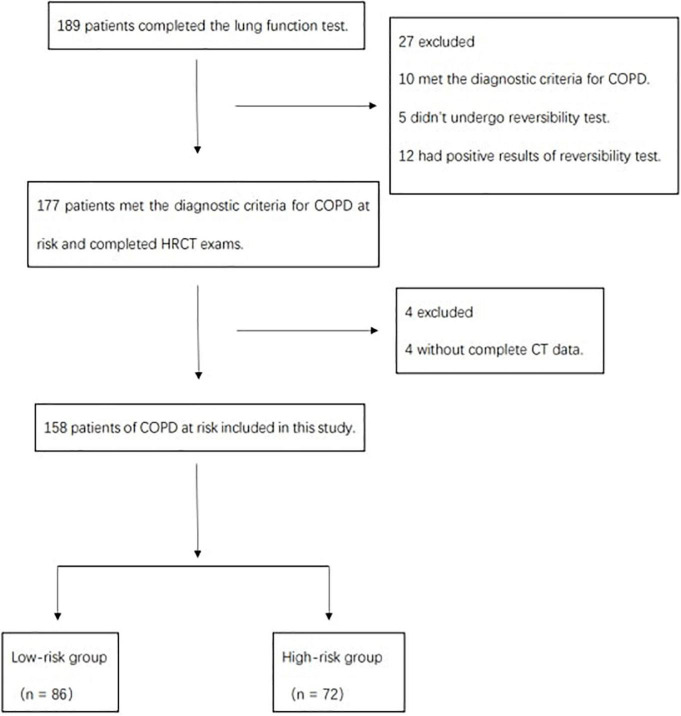
Study flow diagram.

There were 158 participants aged 40–79 years with no prior diagnosis of COPD who were recruited from the Physical Examination Center, Department and Respiratory Medicine and other departments between March 2018 and May 2019 (Figure 1). All of them were older than 40 years, had chronic cough and expectoration, and/or had smoking history and exposurement to dust or chemicals. They underwent respiratory biphasic HRCT, PFTs, and filled out three detailed questionnaires. The interval between PFT and HRCT scan couldn’t exceed 72 h. The risk of developing COPD was determined by the forced expiratory volume in the first second of expiration to forced vital capacity (FEV_1_/FVC): low risk (FEV_1_/FVC ≥ 80%), or high risk (80% > FEV_1_/FVC ≥ 70%) (17, 18).

Exclusion criteria were as follows: asthma, severe pneumonia, tuberculosis, pulmonary fibrosis, or other concomitant chronic diseases, including cardiovascular disease, skeletal muscle dysfunction, metabolic syndrome, osteoporosis, depression, anxiety, and lung cancer.

Medical information and patient characteristics including age, sex, height (m), weight (kg), smoking index, and body mass index (BMI) were obtained from screening questionnaires. All data were stored in an encrypted database.

### Clinical Scores and Questionnaires

All subjects were asked to independently complete three questionnaires, the modified Medical Research Council (mMRC), the chronic obstructive pulmonary disease Assessment Test (CAT), and the clinical chronic obstructive pulmonary disease questionnaire (CCQ). After exhaustive examination, the researchers then recorded the data from the completed questionnaires.

### Spirometry

Spirometry was performed using a Vostro15 portable spirometer according to the ATS/ERS standard. The percentage predicted values were calculated using the equations for Asian adults supplied in the user’s manual. After a 10-min rest, the subject assumed a seated position with feet in full contact with the floor, breathed for three cycles, then forcefully, rapidly, and deeply inhaled through the interface device and suddenly, continuously, and steadily exhaled to reach a maximum amount of breathing. It was necessary for the exhalation process to not be spontaneously interrupted by unsolicited coughing. Measurements were performed in triplicate for each subject, and each curve was coincident, as far as possible, to qualify the PFT, which included FVC, FEV_1_, FEV_1_%prep, and FEV_1_/FVC.

### Chest High-Resolution Computed Tomography Examination and Image Analysis

Prior to HRCT scanning, patients underwent breathing training to optimize the measurement of maximum inspiration. HRCT was performed at suspended full inspiration and expiration using a Gem Energy Spectroscopy CT (Discovery HD750, GE, United States). Scanning parameters were as follows: tube voltage 120 kV, automatic tube current (mA automatic modulation technique), pitch 0.984, slice thickness 5 mm with 1.25 mm reconstruction interval, detector coverage 40 mm, X-ray tube rotation speed 0.6 slice/rotation, DFOV 30 cm, SFOV 50 cm, and a 512 × 512 matrix.

**FIGURE 2:**
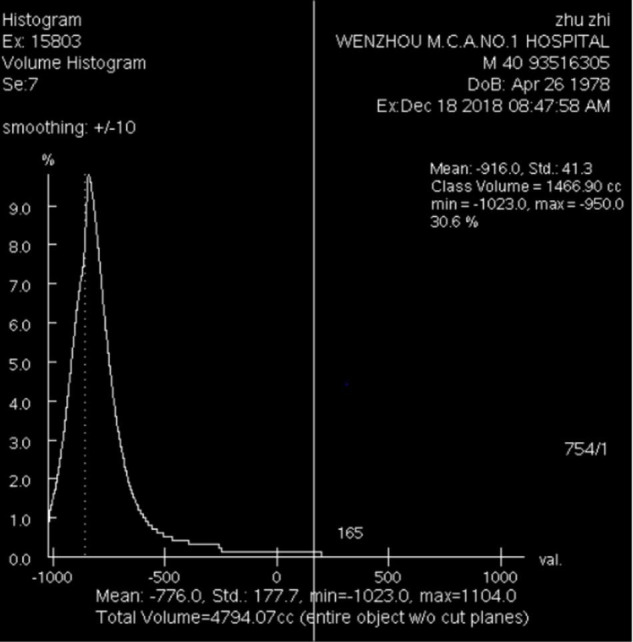
Emphysema volume, total lung volume, emphysema index and MLDins of the entire lung on inspiration CT scan (–950 HUs).

**FIGURE 3:**
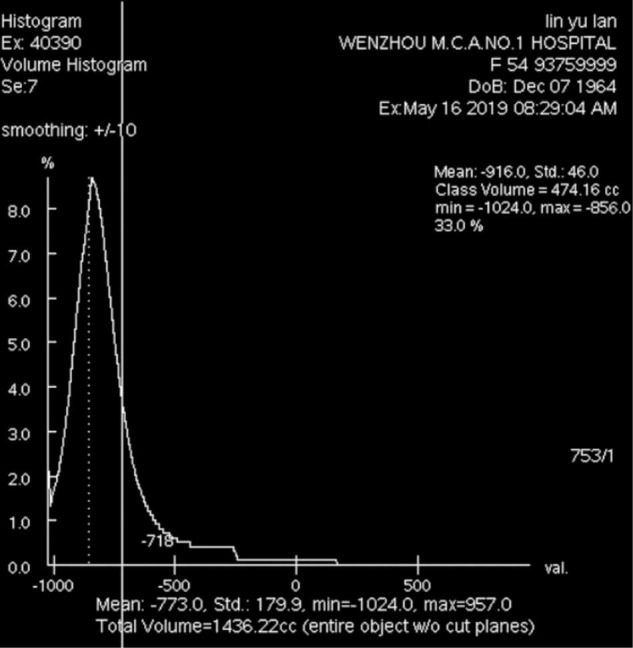
Air trapping volume, total lung volume, air trapping index and MLDexp of the entire lung on expiratory CT scan (–856 HUs).

Quantitative assessments of emphysema were performed using Volume Viewer 11.3 software. We established limits, and the computer program calculated the attenuation as the mean lung density (MLD) of the whole lung (Figures 2, 3). Then, we calculated the total lung area, and the lung area occupied by attenuation values lower than previously fixed thresholds (-950 HUs at inspiration and -856 HUs at expiration) by Lung VCAR (12, 19).

### Statistical Analysis

Statistical analyses were performed using IBM SPSS (Statistical Package for the Social Sciences) Statistics Software (version 22.0, IBM). The measurement data were tested for normality by the Kolmogorov-Smirnov method. Continuous variables of normal distribution are presented as the mean ± standard deviation and were compared by two-sample *t*-test. Continuous variables of skewed distribution are expressed as median values (interquartile ranges) and were compared by the Mann-Whitney U-test. Categorical variables are expressed as a number (%) and were compared by the χ^2^ test or Fisher’s exact test. The correlation between HRCT parameters and pulmonary function parameters was assessed by the Spearman rank correlation test. *p* < 0.05 was considered statistically significant.

## Results

### Baseline Characteristics

The mean age of 158 subjects was 52.18 ± 8.56 years, ranging from 40 to 76 years (Table 1). There were 123 males and 35 females. Overall, 86 (54.4%) participants were under a low risk of developing COPD and 72 were (45.5%) in the high-risk group. There was no statistical difference in BMI, smoking index, or exposure to dust between the two groups. Compared with subjects in the high-risk group, those in the low-risk group had similar scores for mMRC, CAT, and CCQ (*p* = 0.53, *p* = 0.47, and *p* = 0.84, respectively). The FEV_1_/FVC and FEV_1_%prep in the low-risk group were significantly lower than those in the high-risk group (0.82 ± 0.07 vs. 0.79 ± 0.08, 0.92 ± 0.14 vs. 0.86 ± 0.13, *p* < 0.05), while no difference was found in FEV_1_ and FVC (*p* = 0.47 and *p* = 0.93, respectively).

**TABLE 1:** Demographics and baseline characteristics of the study subjects.

Demographics and baseline characteristics	Low-risk group (*n* = 86)	High-risk group (*n* = 72)	*p* value
Age (years)	51.17 ± 7.30	52.73 ± 8.39	0.06
Sex	–	–	0.98
Male	67(77.9%)	56(77.8%)	–
Female	19(22.1%)	16(22.2%)	–
Height (m)	(1.62,1.71)	(1.60,1.71)	0.18
Weight (kg)	65.05 ± 9.07	66.89 ± 9.3	0.21
BMI (kg/m^2^)	23.70 ± 2.99	23.91 ± 2.40	0.63
Exposure to dust (former: never)	22:64	20:52	0.84
Smoking index (pack-years)	(0,22.5)	(0,28.25)	0.90
Clinical questionnaires	–	–	–
mMRC	0.17 ± 0.41	0.22 ± 0.54	0.53
CAT	3.81 ± 4.57	4.36 ± 4.74	0.47
CCQ	1.11 ± 6.19	1.31 ± 6.27	0.84
Spirometry	–	–	–
FEV1 (L)	2.71 ± 0.67	2.64 ± 0.67	0.47
FVC (L)	3.31 ± 0.78	3.31 ± 0.75	0.93
FEV1/FVC	0.82 ± 0.07	0.79 ± 0.08	0.01
FEV1%prep	0.92 ± 0.14	0.86 ± 0.13	0.01

*Data are mean ± standard deviation or median (IQR). p values comparing low-risk group and high-risk group are from two-sample t-test, Mann-Whitney U test, χ^2^or Fisher’s exact test. BMI: body mass index, mMRC: modified British medical research council, CAT: COPD assessment test, CCQ: clinical COPD questionnaire, FEV1: forced expiratory volume in first second, FVC: forced vital capacity, FEV1/FVC: forced expiratory volume in first second/forced vital capacity, FEV1%prep: forced expiratory volume in first second as a percentage of the expected value.*

### Comparison of High-Resolution Computed Tomography Parameters

A comparison of HRCT parameters between the two groups (low-risk group *n* = 86, high-risk group *n* = 72) is shown in Table 2. The mean values of LAA-950ins% in the left lung, right lung, and the entire lung were 1.80 ± 1.73, 5.94 ± 2.00, and 5.92 ± 1.98 in the low-risk group, and 5.69 ± 1.80, 6.52 ± 2.48, and 6.36 ± 1.70 in the high-risk group, respectively. The value of LAA-950ins%, called the emphysema index, showed a difference between two groups only in the left lung (*p* = 0.02), but there was no difference in the right lung or the entire lung (*p* = 0.11 and *p* = 0.15). The mean LAA-856exp% in the left lung, right lung, and the entire lung were 14.75 ± 5.90, 14.36 ± 6.14, and 14.32 ± 6.14 in the low-risk group, and 19.61 ± 6.59, 21.54 ± 6.78, and 21.25 ± 6.63 in the high-risk group, respectively. The value of LAA-856exp%, called the air trapping index, was significantly higher in the high-risk group than in the low-risk group (*p* = 0.00). The value of MLDins in the low-risk group was significantly higher than that in the high-risk group in the left lung (-751.66 ± 29.32 vs. -765.70 ± 38.29, *p* = 0.01) and in the entire lung (-757.51 ± 4.25 vs. -760.40 ± 5.02, *p* = 0.04). The value of MLDexp in the low-risk group was also significantly higher than that in the high-risk group in the left lung (-732.76 ± 44.08 vs. -745.26 ± 35.24, *p* = 0.04), in the right lung (-738.13 ± 36.89 vs. -754.69 ± 30.50, *p* = 0.00), and in the entire lung (-735.86 ± 39.10 vs. -750.49 ± 31.91, *p* = 0.01).

**TABLE 2:** Comparison of high-resolution computed tomography (HRCT) parameters between low-risk group and high-risk group.

	Low-risk group	High-risk group	*p* value
**LAA**_−**950ins**_ **(ml)**			
Left lung	77.92 ± 36.10	90.95 ± 32.09	0.02
Right lung	90.97 ± 39.57	107.04 ± 47.34	0.02
Entire lung	170.08 ± 74.94	195.97 ± 70.93	0.03
**LV_ins_** **(ml)**			
Left lung	1295.87 ± 271.28	1412.11 ± 277.34	0.02
Right lung	1494.76 ± 320.03	1619.00 ± 324.52	0.02
Entire lung	2806.53 ± 600.09	3041.12 ± 590.16	0.02
**LAA**_−**950ins**_ **%**			
Left lung	1.80 ± 1.73	5.69 ± 1.80	0.02
Right lung	5.94 ± 2.00	6.52 ± 2.48	0.11
Entire lung	5.92 ± 1.98	6.36 ± 1.70	0.15
**MLD_ins_**(**HU)**			
Left lung	-751.66 ± 29.32	–765.70 ± 38.29	0.01
Right lung	–747.36 ± 181.63	–761.52 ± 25.28	0.48
Entire lung	–757.51 ± 4.25	–760.40 ± 5.02	0.04
**LAA**_−**856exp**_ **(ml)**			
Left lung	162.34 ± 118.40	265.90 ± 127.46	0.00
Right lung	(61.57,123.62)	(140.49,371.61)	0.00
Entire lung	326.62 ± 209.61	582.05 ± 270.14	0.00
**LV_exp_** **(ml)**			
Left lung	997.28 ± 266.62	1191.13 ± 275.22	0.00
Right lung	1103.39 ± 366.67	1398.92 ± 345.03	0.00
Entire lung	2104.40 ± 561.51	2612.62 ± 606.93	0.00
**LAA**_−**856exp**_%			
Left lung	14.75 ± 5.90	19.61 ± 6.59	0.00
Right lung	14.36 ± 6.14	21.54 ± 6.78	0.00
Entire lung	14.32 ± 6.14	21.25 ± 6.63	0.00
**MLD_exp_** **(HU)**			
Left lung	–732.76 ± 44.08	–745.26 ± 35.24	0.04
Right lung	–738.13 ± 36.89	–754.69 ± 30.50	0.00
Entire lung	–735.86 ± 39.10	–750.49 ± 31.91	0.01

*Data are mean ± standard deviation or median (IQR). p values comparing low-risk group and high-risk group are from two-sample t-test, Mann-Whitney U test, χ^2^ or Fisher’s exact test. LAA_–950ins_: low attenuation areas less than-950HU on inspiratory CT scan, LV_ins_: lung volume on inspiratory CT scan, LAA_–950ins%_: low attenuation areas less than-950HU to lung volume on inspiratory CT scan, MLD_ins_: mean lung density on inspiratory CT scan, HU: housfield unites, LAA_–856exp_: low attenuation areas less than-856HU on expiratory CT scan, LV_exp_: lung volume on expiratory CT scan, LAA_–856exp%_: low attenuation areas less than-856HU to lung volume on expiratory CT scan, MLD_exp_: mean lung density on expiratory CT scan.*

### Correlations Between Quantitative Measurements and Pulmonary Function Parameters

The correlations of various quantitative HRCT parameters at suspended full inspiration with the pulmonary function test variables are shown in Table 3. The LAA-950ins% showed a correlation with the FEV_1_/FVC (*r* = –0.33 in the left lung, *r* = –0.22 in the right lung, *r* = –0.26 in the entire lung, *p* < 0.05), a correlation with FEV_1_ only in the left lung (*r* = –0.19, *p* = 0.04), and no correlation with FVC or FEV_1_%prep. TheLAA-950ins also showed a correlation with the FEV_1_/FVC (*r* = –0.33 in the left lung, *r* = –0.22 in the right lung, *r* = –0.26 in the entire lung, *p* < 0.05), but no correlation with FEV_1_, FVC, or FEV_1_%prep. TheLVins showed a statistical correlation with FVC (*r* = 0.23 in the left lung, *r* = 0.19 in the right lung, *r* = 0.21 in the entire lung, *p* < 0.05), but no correlation with FEV_1_, FEV_1_/FVC, or FEV_1_%prep. The MLDins in the left lung and in the entire lung showed a correlation with the FEV_1_/FVC (*r* = 0.20 in the left lung and *r* = 0.21 in the entire lung, *p* < 0.05), but the MLDins in the right lung had no correlation with the FEV_1_/FVC (*p* = 0.06). There was no statistical correlation between the MLDins and three other physiologic parameters, including FEV_1_, FVC, and FEV_1_%prep (*p* > 0.05).

**TABLE 3:** The correlation between quantitative high-resolution computed tomography (HRCT) parameters at inspiration and pulmonary function parameters.

		FEV_1_		FVC		FEV_1_/FVC		FEV_1_%prep	
		*r*	*p*	*r*	*p*	*r*	*p*	*r*	*p*
Left lung	LAA_−950ins_	–0.07	0.46	0.02	0.80	–0.33**	0.00	–0.12	0.19
	LV_ins_	0.16	0.09	0.23*	0.01	–0.23*	0.01	–0.07	0.47
	LAA_−950ins_%	–0.19*	0.04	–0.11	0.26	–0.33**	0.00	–0.12	0.18
	*MLD*_*ins*_	0.07	0.48	0.01	0.91	0.20*	0.03	0.05	0.57
Right lung	LAA_−950ins_	–0.02	0.81	0.05	0.60	–0.22*	0.02	–0.09	0.35
	LV_ins_	0.15	0.12	0.19*	0.04	–0.12	0.18	–0.08	0.42
	LAA_−950ins_%	–0.15	0.12	–0.08	0.42	–0.22*	0.02	–0.09	0.35
	*MLD*_*ins*_	0.02	0.84	–0.03	0.76	0.17	0.06	0.02	0.81
Entire lung	LAA_−950ins_	0.00	0.99	0.08	0.39	–0.26**	0.01	–0.09	0.34
	LV_ins_	0.15	0.10	0.21*	0.02	–0.18	0.05	–0.08	0.40
	LAA_−950ins_%	–0.12	0.20	–0.05	0.63	–0.26**	0.01	–0.11	0.26
	*MLD*_*ins*_	0.07	0.45	0.01	0.94	0.21*	0.02	0.05	0.57

**p < 0.01, *p < 0.05. FEV1: forced expiratory volume in first second, FVC: forced vital capacity, FEV1/FVC: forced expiratory volume in first second/forced vital capacity, FEV1%prep: forced expiratory volume in first second as a percentage of the expected value, LAA_–950ins_: low attenuation areas less than-950HU on inspiratory CT scan, LV_ins_: lung volume on inspiratory CT scan, LAA_–950ins%_: low attenuation areas less than-950HU to lung volume on inspiratory CT scan, MLD_ins_: mean lung density on inspiratory CT scan.

The LAA-856exp% showed a correlation with FEV_1_, FEV_1_/FVC, and FEV_1_%prep (*p* < 0.05) in Table 4. Among them, the most optimal correlation between LAA-856exp% was found with FEV_1_/FVC (*r* = –0.33 in the left lung, *r* = –0.23 in the right lung, and *r* = –0.28 in the entire lung). No useful correlation was found between the LAA-856exp% and FVC. Both LAA-856exp and LVexp showed statistical correlation with FEV_1_/FVC (*r* = –0.32 in the left lung, *r* = –0.23 in the right lung, *r* = –0.28 in the entire lung, and *r* = –0.29 in the left lung, *r* = –0.17 in the right lung, *r* = –0.25 in the entire lung, respectively, *p* < 0.05). The LAA-856exp% and LVexp also showed correlation with FEV_1_%prep (*r* = –0.24 in the left lung, *r* = –0.26 in the right lung, *r* = –0.25 in the entire lung and *r* = –0.25 in the left lung, *r* = –0.24 in the right lung, *r* = –0.27 in the entire lung, respectively, *p* < 0.05), but neither of them correlated with FEV_1_or FVC. For the MLDexp, positive correlations were found with FEV_1_/FVC (*r* = 0.31 in the left lung, *r* = 0.25 in the right lung and *r* = 0.29 in the entire lung, *p* < 0.01), while no statistical correlation was found with FEV_1_, FVC, and FEV_1_%prep (*p* > 0.05).

**TABLE 4:** The correlation between quantitative high-resolution computed tomography (HRCT) parameters at expiration and pulmonary function parameters.

		FEV_1_		FVC		FEV_1_/FVC		FEV_1_%prep	
		*r*	*p*	*r*	*p*	*r*	*p*	*r*	*p*
Left lung	LAA_−856exp_	–0.13	0.14	–0.09	0.34	–0.32**	0.00	–0.24**	0.01
	LV_exp_	–0.06	0.52	–0.01	0.92	–0.29**	0.01	–0.25**	0.01
	LAA_−856exp%_	–0.19*	0.04	–0.15	0.11	–0.33**	0.00	–0.24**	0.01
	*MLD*_*exp*_	–0.06	0.50	–0.14	0.13	0.31**	0.00	0.03	0.73
Right lung	LAA_−856exp_	–0.16	0.08	–0.13	0.16	–0.23*	0.01	–0.26**	0.00
	LV_exp_	–0.08	0.38	–0.06	0.50	–0.17*	0.03	–0.24*	0.01
	LAA_−856exp%_	–0.20*	0.03	–0.17	0.08	–0.23*	0.01	–0.25**	0.01
	*MLD*_*exp*_	–0.06	0.49	–0.13	0.15	0.25**	0.00	0.04	0.69
Entire lung	LAA_−856exp_	–0.14	0.14	–0.10	0.29	–0.28**	0.00	–0.25**	0.01
	LV_exp_	–0.09	0.34	–0.05	0.59	–0.25**	0.01	–0.27**	0.00
	LAA_−856exp%_	–0.20*	0.03	–0.16	0.08	–0.28**	0.00	–0.24**	0.01
	*MLD*_*exp*_	–0.08	0.41	–0.15	0.10	0.29**	0.00	0.02	0.80

**p < 0.01, *p < 0.05. FEV1: forced expiratory volume in first second, FVC: forced vital capacity, FEV1/FVC: forced expiratory volume in first second/forced vital capacity, FEV1%prep: forced expiratory volume in first second as a percentage of the expected value, LAA_–856exp_: low attenuation areas less than-856HU on expiratory CT scan, LV_exp_: lung volume on expiratory CT scan, LAA_–856exp%_: low attenuation areas less than-856HU to lung volume on expiratory CT scan, MLD_exp_: mean lung density on expiratory CT scan.

## Discussion

Our observational study on 158 patients who were at risk of developing COPD demonstrates that HRCT measurements of emphysema index and air trapping index were negatively correlated with FEV_1_/FVC from PFT, whereas the mean expiratory lung density showed a positive correlation. Patients in the high-risk group exhibited a significantly higher air trapping index but lower mean expiratory lung density than those in the low-risk group.

COPD is a chronic inflammatory airway disease that is characterized by airflow limitations that are not fully reversible (20). At the early stage of the disease, patients are often asymptomatic or exhibit only mild chronic cough or dyspnea (21, 22). However, as the disease progresses, patients may experience chest pain, expectoration, fatigue, weight loss, and can also develop acute lower respiratory infections, cardiovascular disease, or lung cancer that continue to place an enormous burden on society (23, 24). According to the latest research, the prevalence of COPD in China is continually increasing, and the subgroup of patients over 40 years of age has nearly 99 million people, accounting for 13.7% (25). Therefore, early detection and timely management are imperative.

There has been interest in the diagnostic value of HRCT for COPD, and its combined use with advanced postprocessing software will provide important clinical applications for COPD. Several studies (25–27) have established the relationship between pulmonary function parameters such as FEV_1_/FVC, RV/TLC, and CT findings. Some CT scans have been previously used to classify the severity in COPD patients. However, articles discussing CT findings in patients at risk of developing COPD are scarce. The present study shows that the HRCT EI, ATI, and the mean expiratory lung density are all independent diagnostic factors for patients at risk of developing COPD, and represent independent imaging biomarkers (28, 29).

We find from Table 1 that compared with the low-risk group, there was no statistically significant difference in the clinical features (age, sex, BMI categories, dust exposure, and smoking index) and the clinical symptom scores (mMRC, CAT, and CCQ) for the high-risk group. However, the differences in the forced expiratory volume in the first second/forced vital capacity and forced expiratory volume in the first second as a percentage of the expected value showed significant difference (*p* < 0.01) between the two groups. This suggests that based on behavioral observation and recordings, the severity of COPD in the different states was indistinguishable. These results were in agreement with the findings of Sunmin Kim et al. (30). Their conclusion shows that the choice of symptom scale can alter the group assignment of COPD. The clinical features and the clinical symptom scores can be used to assist in gauging the condition, but there were limitations to the evaluation at the individual level. It is clear that additional indicators need to be comprehensively assessed in combination.

Comparing the CT parameters of the low-risk and high-risk COPD groups, the CT images of patients from the high-risk group exhibit certain clinical characteristics and typical imaging features. Higher lung volume and emphysema index, and lower mean lung density in the inspiratory phase and expiratory phase were present. This result demonstrates that in COPD high-risk patients, pulmonary hyperinflation and lung volume increased. Patients in the high-risk group have a significantly higher air trapping index but lower mean expiratory lung density as compared to the low-risk group. As for the emphysema index and the mean inspiratory lung density, this trend in the high and low-risk group was not statistically significant. Therefore, it is suggested that the air trapping index was more accurate at predicting the risk state of COPD and pulmonary function decline than the emphysema index. In other words, using HRCT imaging for the study of lung pathologies in the early stage of the disease, the CT scans obtained at expiration were more informative than those at inspiration. In previous studies, the choice of an end inspiration or end expiration image remained controversial (31). Some demonstrated the greater diagnostic sensitivity of inspiratory lung volumes for COPD (32). CT measurements of airway dimensions and emphysema are useful and complementary in the evaluation of the lung with COPD.

Our study on the correlation between CT parameters and pulmonary function shows that the left, right, and bilateral lung emphysema index and air trapping index were significantly inversely correlated with FEV_1_/FVC. This indicates that the emphysema index theoretically has the potential to use HRCT to assess evidence of emphysema and small airway disease. Our results indicate that the presence of increasing lung volume, persistent airflow limitation, and air trapping can also be present in those with undiagnosed COPD, which can in turn lead to pulmonary function changes. In our study, we also found that the left, right, and bilateral lung mean expiratory lung density showed significantly positive correlation with FEV_1_/FVC. This is in accordance with the conclusion arrived at by previously published studies (33–35). There was high predictive value of CT density parameters for detecting pulmonary ventilation.

The aim of our study was to assess the HRCT and pulmonary function status of people at risk of COPD and provide a useful supplement for the early and comprehensive assessment of the disease. Therefore, the key population we focus on in this study was low-risk and high-risk patients of undiagnosed COPD. Since many studies have proved that HRCT and pulmonary function test can be used to evaluate the severity of COPD patients (36–38), we consider that these two indicators have certain significance as an assessment of the risk of COPD.

Our study has several limitations. First, the study was conducted in a single center in China and was carried out at an outpatient clinic where patients had morbid conditions, and thus, inpatients were not included in the study. Second, the study population was relatively small and predominantly male. The results might be different in a larger population. Third, we did not enroll patients with confirmed COPD as controls, and therefore, the extrapolation of these findings to COPD cohorts must be performed with care. Last, no follow-up of the patients was performed.

## Conclusion

In summary, our original study demonstrates that the emphysema index, air trapping index and the mean expiratory lung density obtained by HRCT show significant correlation with FEV_1_/FVC, which can be used to assess the pulmonary function status of people at risk of COPD, and as an indicator for distinguishing between high and low-risk for patients of COPD and provide a useful supplement for the early and comprehensive assessment of the disease.

## Data Availability Statement

The raw data supporting the conclusions of this article will be made available by the authors, without undue reservation.

## Ethics Statement

The studies involving human participants were reviewed and approved by Ethics committee of The First Affiliated Hospital of Wenzhou Medical University. The patients/participants provided their written informed consent to participate in this study. Written informed consent was obtained from the individual(s) for the publication of any potentially identifiable images or data included in this article.

## Author Contributions

LY, XH, and YiW conceived and designed the study, responsible for the integrity and accuracy of the data, and had full access to the study. RL and HJ contributed to drafting and writing this manuscript. MX took responsibility for obtaining written consent from patients, obtaining ethical approval, collecting samples, and confirming data accuracy. PS, YaW, and DY made substantial contributions to data acquisition, analysis, and interpretation. All authors had strictly revised the manuscript, agreed to be responsible for all aspects of the work, and finally approved the version to be published.

## Conflict of Interest

The authors declare that the research was conducted in the absence of any commercial or financial relationships that could be construed as a potential conflict of interest.

## Publisher’s Note

All claims expressed in this article are solely those of the authors and do not necessarily represent those of their affiliated organizations, or those of the publisher, the editors and the reviewers. Any product that may be evaluated in this article, or claim that may be made by its manufacturer, is not guaranteed or endorsed by the publisher.

## Abbreviations

COPD: chronic obstructive pulmonary disease
CT: computed tomography
HRCT: high-resolution computed tomography
HU: housfield unites
PFTs: pulmonary function tests
BMI: body mass index
FVC: forced vital capacity
FEV1: forced expiratory volume in first second
FEV1%: prep forced expiratory volume in first second as a percentage of the expected value
FEV1/FVC: forced expiratory volume in first second/forced vital capacity
LAA: low attenuation areas
LAA_–856exp_: low attenuation areas less than-856HU on expiratory CT scan
LAA_–950ins_: low attenuation areas less than-950HU on inspiratory CT scan
LAA_–856exp%_: low attenuation areas less than-856HU to lung volume on expiratory CT scan
LAA_–950ins%_: low attenuation areas less than-950HU to lung volume on inspiratory CT scan
MLD: mean lung density
MLD_*exp*_: mean lung density on expiratory CT scan
MLD_*ins*_: mean lung density on inspiratory CT scan
LV_*ins*_: lung volume on inspiratory CT scan
LV_*exp*_: lung volume on expiratory CT scan
mMRC: modified British medical research council
CAT: COPD assessment test
CCQ: clinical COPD questionnaire
EI: emphysema index
ATI: air trapping index
RV/TLC: residual volume/total lung capacity.
